# Neutrophil Extracellular Trap Formation Model Induced by Monosodium Urate and Phorbol Myristate Acetate: Involvement in MAPK Signaling Pathways

**DOI:** 10.3390/ijms26010143

**Published:** 2024-12-27

**Authors:** Chenxi Wu, Xinru Xu, Yueyue Shi, Fenfen Li, Xiaoxi Zhang, Yan Huang, Daozong Xia

**Affiliations:** 1 School of Pharmaceutical Sciences, Zhejiang Chinese Medical University, Hangzhou 310053, China; 202011014011089@zcmu.edu.cn (C.W.); xuxinru0607@163.com (X.X.); 202211014011060@zcmu.edu.cn (Y.S.); ncusklifenfen@163.com (F.L.); 202111113911038@zcmu.edu.cn (Y.H.); 2 Academy of Chinese Medical Sciences, Zhejiang Chinese Medical University, Hangzhou 310053, China; 20201059@zcmu.edu.cn

**Keywords:** neutrophil extracellular traps, monosodium urate, phorbol myristate acetate, MAPK signaling pathway

## Abstract

Neutrophil extracellular traps (NETs) formation is a key process in inflammatory diseases like gout, but the underlying molecular mechanisms remain incompletely understood. This study aimed to establish a model to examine the formation of NETs induced by monosodium urate (MSU) and phorbol 12-myristate 13-acetate (PMA) and to elucidate their molecular pathways. Laser confocal microscopy was used to visualize NET formation, while flow cytometry was employed to detect reactive oxygen species (ROS) production. The microstructure of neutrophils was observed by transmission electron microscopy, and the expression of key proteins was determined by Western blotting. Additionally, the effect of various inhibitors targeting the MAPK signaling pathway on NET formation was evaluated. They include the Ras inhibitor Salirasib, Raf inhibitor Vemurafenib, ERK inhibitor PD98059, and p38 MAPK inhibitor SB203580, as well as NADPH oxidase inhibitor DPI and neutrophil elastase inhibitor Alvelestat. The results showed that MSU and PMA triggered significant NET formation, which was accompanied by increased ROS levels, lactate dehydrogenase release, dsDNA, and IL-8. Notably, selective MAPK pathway inhibitors and DPI and Alvelestat, except for SB203580, effectively down-regulated these indicators. These data indicated that the activation of a signaling pathway involving Ras-Raf-ERK, which is dependent on ROS, is crucial for the induction of NET formation by MSU and PMA. Given the involvement of NETs in multiple pathologies, our findings could potentially serve as molecular targets for the intervention and treatment of crystal-related diseases, especially for gout.

## 1. Introduction

Neutrophil extracellular traps (NETs) are DNA-based web-like structures formed by neutrophils in response to various stimuli, a process known as NETosis [1]. These NETs are decorated with proteins such as neutrophil elastase (NE) and myeloperoxidase (MPO), which can effectively capture and kill a variety of microorganisms, including bacteria, viruses, and fungi [2,3,4]. However, excessive release of NETs disrupts immune homeostasis, leading to inflammation and tissue damage, thereby contributing to various diseases [5,6,7]. For example, excessive NETs can lead to diseases such as diabetes and its complications, including diabetic retinopathy [8]. Additionally, other studies [9,10] have shown that endogenous crystals, such as cholesterol, monosodium urate (MSU) crystals, and calcium carbonate crystals, can induce NETosis via the activation of the RIPK1-RIPK3-MLKL signaling pathway, leading to inflammatory conditions such as gout and pancreatitis. Additionally, pro-inflammatory cytokines like IL-17A, TNF-α, and IL-1β can promote NETosis through NADPH oxidase (NOX)-dependent mechanisms [10,11]. Cancer-associated fibroblasts-derived amyloid-beta can trigger NOX-dependent pro-tumorigenic NET release via CD11b, while activated platelets can also induce NETosis by delivering lipopolysaccharide and other signaling molecules [10,12]. Therefore, the dual roles of NETs in autoimmune diseases, cancer, and inflammatory disorders have attracted significant attention.

Reactive oxygen species (ROS) are known to be key initiators of NETosis, with the activation of NOX being a critical factor in ROS formation [13,14,15]. Ansari et al. [16] demonstrated that NOX-dependent ROS serve as the final and crucial determinant of pathologic NET production, which exacerbate cerebral vascular thrombotic inflammation. Li et al. [17] found that *Streptococcus uberis* induces NOX-dependent NET formation, which damages mammary epithelial cells and barriers, contributing to the development of mastitis. Schorn et al. [18] showed that MSU crystals induce NETosis in a ROS-dependent manner in vitro and that various antioxidants can partially inhibit MSU crystal-induced NETosis. Studies [19,20] have shown that ROS can induce NETosis through the MAPK signaling pathway and the peptidyl arginine deiminase 4 (PAD4)-mediated citrullination of histone arginine residues, thereby reducing the DNA-binding capacity of neutrophils. Additionally, ROS facilitate the translocation of MPO and NE from the cytoplasm to the nucleus, leading to histone disruption, chromatin decondensation, and, ultimately, NETosis [21,22]. Awasthi et al. [23] reported that oxidized low density lipoprotein, an independent risk factor for various acute or chronic inflammatory diseases, triggers NET formation via the TLR-PKC-IRAK-MAPK signaling pathway and by activating NOX to produce ROS. Cao et al. [24] discovered that magnesium hydride significantly inhibits phorbol 12-myristate 13-acetate (PMA)-induced ROS production and the expression of key proteins, thereby suppressing NET formation through a ROS/MAPK/PAD4-dependent mechanism and reducing NET-related intestinal barrier damage. These findings indicate that ROS regulate NET formation via the MAPK signaling pathway, thereby influencing the pathogenesis of inflammatory diseases such as gout.

Gout is a metabolic disease caused by a disorder of purine metabolism, which leads to the accumulation of uric acid in joint tissues and the formation of MSU deposits; it is also termed gouty arthritis (GA) [25]. NETs are believed to play a role in the pathogenesis of GA. During the early stage of a GA attack, neutrophils are continuously recruited to the inflamed joint area. Activated neutrophils undergo NETosis and release inflammatory mediators upon the uptake of MSU crystals [26,27]. As local joint inflammation worsens, a large number of inflammatory factors are produced and enter the circulatory system, resulting in a full-scale GA attack [28]. Mitroulis et al. [29] demonstrated that 250 μg/mL MSU can induce neutrophils to form NETs, a process associated with IL-1β and autophagy signaling. The generation of NETs in response to 1 mg/mL MSU requires the involvement of TAK1 and Syk upstream, as reported by Tatsiy et al. [30]. Garcia’s study [31] indicated that in crystal-induced arthritis, the release of NETs is independent of the number of white blood cells and the type of crystals but is dependent on the quantity of crystals present. These findings suggest that the mechanisms underlying NET production in GA remain unclear and warrant further investigation to gain deeper insights into the multifaceted roles of NETs in GA. This will lay the foundation for developing new therapeutic strategies. Additionally, we note that there is considerable variability in the doses of MSU used to induce NETs in vitro, as shown by Garcia’s study [31], where the release of NETs is closely related to the number of crystals. This variability introduces instability into the models, thereby introducing uncertainty into the studies of NET formation mechanisms.

Several studies have shown that MSU can trigger the release of ROS; for example, Zeng et al. [32] and Yin et al. [33] found that MSU can induce the production of ROS in RAW264.7 cells, and MSU-induced gouty mice exhibit significant oxidative stress with elevated levels of ROS. PMA, as an NOX activator, activates NOX and generates ROS, and it is also a potent inducer of NETs in vitro and can be used as an appropriate mimic of physiological agonists [34,35]. The limiting solubility of MSU is 6.8 mg/dL (approximately 400 μM) [18,36,37]. Neutrophils can form NETs using MSU alone, but the required dose is generally higher (up to 1 mg/mL) [30,38], and the dose is not uniform [18,36,39,40]. However, high concentrations of MSU may also affect cellular functions such as cellular phagocytosis and respiration. Meanwhile, the mechanisms by which different doses of MSU induce the formation of NETs vary. NETs generated by low doses of MSU (200 μg/mL) exhibit pro-inflammatory effects [18], whereas NETs produced by high doses of MSU (1 mg/mL) display aggregated structures with anti-inflammatory properties [38]. In crystal-induced diseases, the concentration of pathogenic substances often exceeds their solubility limits. Therefore, in this study, we selected an MSU concentration of 500 μM. A single lower concentration of crystals (e.g., MSU) will facilitate the understanding of the pathological state at the onset of disease (such as GA), and this concentration is consistent with our previous research [41,42].

Taken together, this study aims to establish and evaluate a NET model induced by MSU combined with PMA. This effort will enhance our understanding of NET formation and provide a more consistent cellular model for future investigations.

## 2. Results

### 2.1. Neutrophils Purity

**Figure 1:**
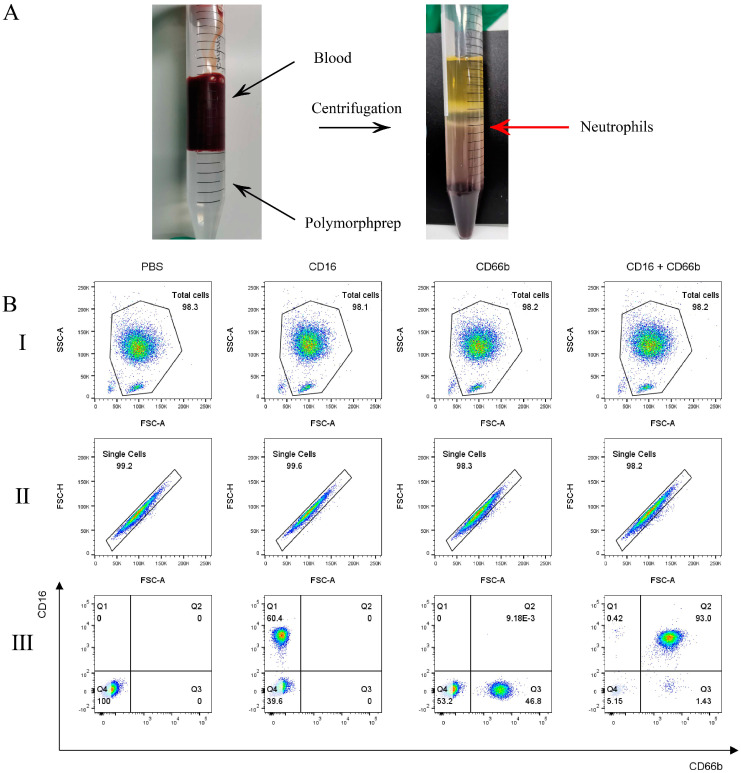
Extraction, isolation, and identification of human neutrophils: (**A**) Human peripheral blood was collected using an anticoagulant tube containing EDTA-K2, and human neutrophils were isolated in accordance with the instructions of Polymorphprep^TM^. The red arrows point to the neutrophil layer. (**B**) Cytometric markers and gating strategies were used to analyze the human neutrophil populations. (I) Cell debris was excluded using FSC-A/SSC-A plots. (II) The gate to exclude cell aggregates in the FSC-A/FSC-H diagram. (III) Neutrophils were identified using PE-CD16 and FITC-CD66b markers, achieving a purity of over 93%.

PolymorphPrep^TM^ was used to extract and isolate neutrophils (Figure 1A). CD16 and CD66b were used to label neutrophils for assessing cell purity. Flow cytometry analysis of PE-CD16+ and FITC-CD66b+ cells indicated that the purity reached 93% (Figure 1B). The result showed that a high purity neutrophil could be obtained using PolymorphPrep^TM^, making them suitable for subsequent experiments.

### 2.2. NETs Are Formed by MSU and PMA-Induced Neutrophil

**Figure 2:**
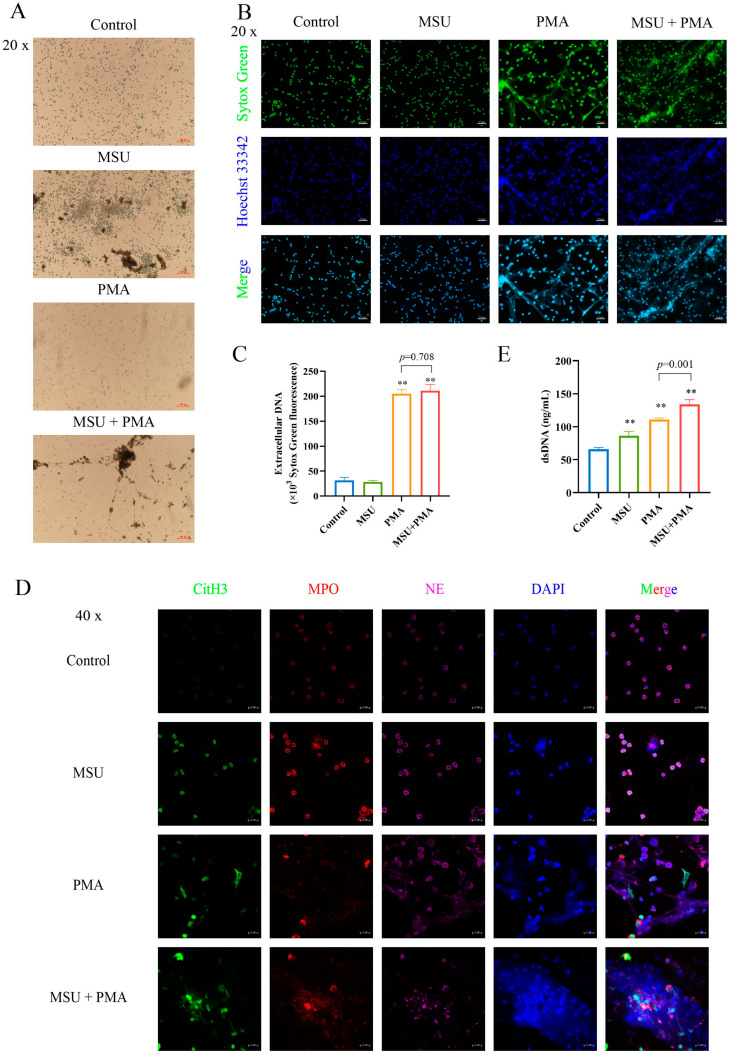
MSU and PMA induces NET formation: (**A**) The morphological changes of cells treated with MSU and/or PMA were observed under light microscope. Scale bars, 100 μm. (**B**) Human neutrophils were seeded in 12-well plates containing L-polylysine slides. After treatment with MSU and/or PMA, the cells were fixed 4% paraformaldehyde. The formation of NETs was observed using Sytox Green dead cell nucleic acid dye (green) and Hoechst 33342 dye (blue). Scale bars, 50 μm. (**C**) The generation of NETs was quantified by Sytox Green dye at Ex/Em: 502/525 nm using a fluorescence microplate reader, *n* = 3. (**D**) Human neutrophils (2 × 10^5^ cells/well) were seeded in 12-well plates containing L-polylysine slides. Triton X-100 was permeabilized and 5% BSA was blocked. Then, samples were stained with these primary antibodies: rabbit Histone H3 (citrulline R2 + R8 + R17) (CitH3) antibody (1:400, green), goat MPO antibody (1:200, red), mouse NE antibody (1:200, pink), they incubated in a wet box at 4 °C overnight. Secondary antibodies were then blocked for 1 h at room temperature and DAPI (blue) for 10 min. Compared to unstimulated neutrophils, MSU and/or PMA-stimulated cells show membrane rupture and release of cellular contents (such as MPO and NE), appearing as filamentous structures, with some histones undergoing citrullination. Scale bars, 20 μm. (**E**) Neutrophils (1 × 10^6^ cells/mL) were seeded in a 6-well plate. After various treatments, the supernatants were collected. The supernatants were then centrifuged, and the resulting supernatants were mixed with PicoGreen reagent and incubated at room temperature in the dark for 5 min. The fluorescence intensity was measured at Ex/Em: 480/520 nm, *n* = 4. The data were presented as mean ± SEM with ** *p* < 0.01 vs. Control group. MSU, monosodium urate; PMA, phorbol 12-myristate 13-acetate; NET, neutrophil extracellular trap; CitH3, citrulline Histone H3; MPO, myeloperoxidase; NE, neutrophil elastase.

First, we observed the morphology of cells treated with MSU and/or PMA using an inverted fluorescence microscope (Figure 2A). Neutrophils without stimulation exhibited a bright, round shape with an intact cell membrane. After treatment with different stimuli, the cell morphology changed distinctly. MSU at 500 μM did not significantly alter the morphology of neutrophils, while PMA at 50 nM resulted in flattened or irregularly shaped cells, with only a few remaining round. Following combined stimulation with MSU and PMA, the cells appeared flat and dull.

Next, we assessed the formation of NETs using Sytox Green, a dead cell nucleic acid dye specifically designed to label NETs, and Hoechst 33342, a nuclear dye. The nuclei of unstimulated neutrophils appeared horseshoe-shaped or multinucleated, with a clear nuclear interface. After stimulation with 500 μM MSU, the nuclei of neutrophils did not change significantly. In contrast, 50 nM PMA treatment led to significant changes in the morphology of neutrophils. The cell volume was significantly larger, most of the horseshoe-shaped nuclei disappeared, and an obvious reticular structure appeared in the field of view, which means many filaments appeared near the cells. However, after combined stimulation with MSU + PMA, cells aggregated and released chromatin, resulting in a more pronounced network structure (Figure 2B).

Finally, we quantified the generation of NETs. Sytox Green is a green nucleic acid dye that can easily penetrate the plasma membrane of damaged cells but not live ones. This property was utilized to quantify extracellular NETs by measuring the fluorescence intensity of Sytox Green. The fluorescence intensity produced by 500 μM MSU alone did not change significantly compared with the Control group, whereas the fluorescence intensity produced by 50 nM PMA and 50 nM PMA + 500 μM MSU showed significant changes (Figure 2C).

### 2.3. MSU and PMA Promote the Release of the Intracellular Components of Neutrophils

To observe the distribution of key proteins in NETs, confocal laser microscopy was used to analyze the localization of CitH3, MPO, and NE (Figure 2D). In unstimulated neutrophils, MPO and NE were evenly distributed throughout the cytoplasm and were not released from the cell. After MSU + PMA combined stimulation, the cell membrane and nucleus were ruptured, leading to the release of proteases and granular enzymes. Additionally, some histones were citrullinated, and the reticular structure was obvious.

Since dsDNA is a key component of NETs, the content of dsDNA in the supernatant was measured. The dsDNA content was significantly increased in the MSU and/or PMA group compared to the Control group (Figure 2E).

### 2.4. The Structure of Neutrophils Was Changed by MSU and PMA

**Figure 3:**
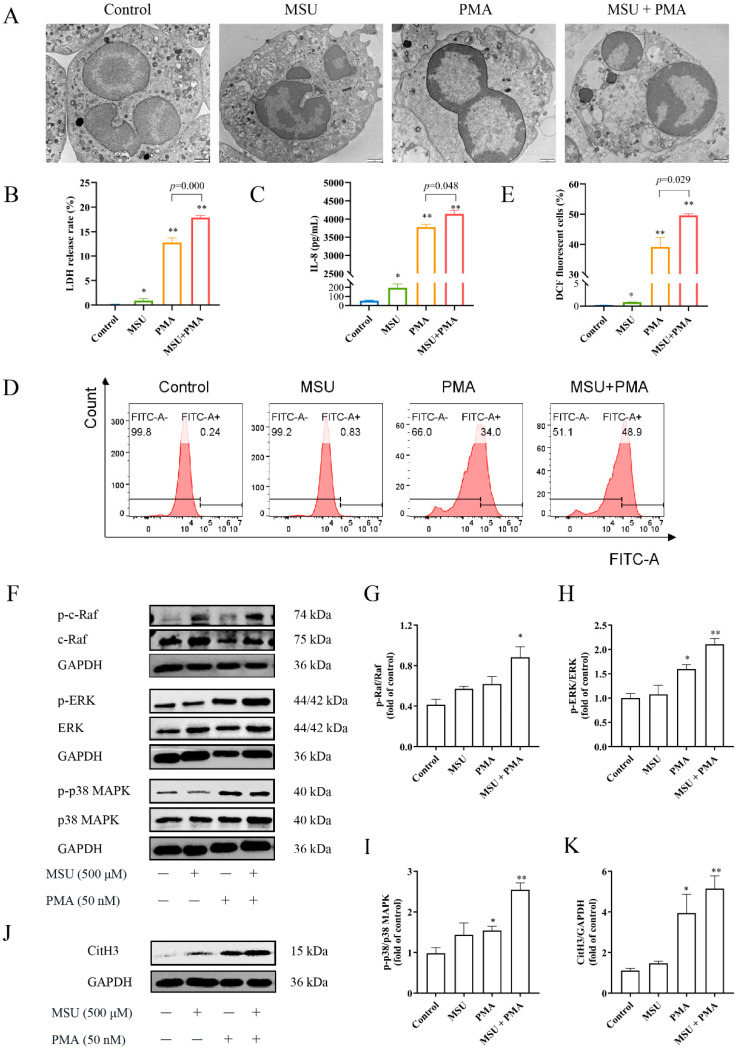
MSU and PMA promote ROS production and activate the MAPK signaling pathway. (**A**) After treatment with MSU and/or PMA, the human neutrophils were fixed with 2.5% glutaraldehyde, followed by staining, dehydration, embedding, and sectioning. TEM was used to observe the internal structure of NETs. Neutrophils stimulated by MSU and/or PMA exhibit incomplete or ruptured cell membranes and nuclear envelopes, with vacuoles appearing around the nuclear envelope. There is also chromatin condensation, a reduction in the number of organelles, and, in particular, a significant decrease in the endoplasmic reticulum and mitochondria. TEM images of human neutrophils exposed to MSU and/or PMA. (Scale bar: 1 μm). (**B**) 5 × 10^4^ cells/well neutrophils were stimulated with 500 μM MSU and 50 nM PMA. When the specified time was reached, 120 μL supernatant was reacted with 60 μL of the LDH detection working solution, and the LDH release (cell death rate) was detected at dual wavelengths (490/600 nm), *n* = 8. (**C**) The cell supernatants were collected to determinate the IL-8 content, *n* = 3. (**D**) All cells were added DCFH-DA (1:1000) prepared in serum-free medium and incubated for 20 min. Then, after different treatments of neutrophils, cells were collected by centrifugation and intracellular ROS fluorescence intensity were detected by Beckman flow cytometry. The solid line represents the proportion of cells that are DCFH-DA positive (FITC-A +). (**E**) The three results of (**D**) were counted. (**F**–**K**) The key proteins of the MAPK signaling pathway and CitH3 in human neutrophils were detected by Western blotting. The data were presented as mean ± SEM with * *p* < 0.05 or ** *p* < 0.01 vs. Control group. ROS, reactive oxide species; TEM, transmission electron microscopy; LDH, lactate dehydrogenase.

Subsequently, TEM was utilized to observe microstructure changes in neutrophils upon stimulation with different stimuli (Figure 3A). In its normal state, without any stimulation, the neutrophil cell membrane appears intact without signs of rupture or fragmentation. The nucleus typically shows 2–3 horseshoe-shaped or irregularly shaped, with a clearly visible nuclear membrane and non-condensed chromatin. The cytoplasm contains numerous lysosomes and mitochondria, while the endoplasmic reticulum and Golgi apparatus are less prominent, and there are abundant granules. Upon MSU + PMA stimulation, the cell membrane completely lysed, releasing all intracellular contents. The nucleus also ruptured, releasing chromatin into the cytoplasm and extracellular space. Occasionally, remnants of the cell membrane were observed, but the endoplasmic reticulum and mitochondria were rarely seen. The space between the inner and outer nuclear membranes expands, with part of the nuclear envelope already incomplete. Some cytoplasmic vacuoles were also noted.

The release of LDH is regarded as an important indicator of cell membrane integrity. As expected, both 500 μM MSU and 50 nM PMA stimulation induced LDH release in neutrophils, with PMA exhibiting a stronger capacity to disrupt cell membranes. Moreover, the combined stimulation with 50 nM PMA and 500 μM MSU resulted in a cell mortality rate exceeding 15%.

### 2.5. MSU and PMA Induce Neutrophils to Release Chemokines and Produce ROS

IL-8, a chemotactic inflammatory cytokine, showed no significant change after 500 μM MSU stimulation; however, its levels significantly increased following stimulation with MSU and PMA (Figure 3C). ROS is an essential prerequisite for the generation of NETs. While 500 μM MSU produced a small amount of ROS, the combination of MSU and PMA stimulated neutrophils to generate approximately 50% of the ROS (Figure 3D,E).

### 2.6. The NETs Produced by MSU and PMA Is Related to MAPK Signaling Pathway

Excessive ROS can reduce the DNA-binding capacity of neutrophils, which is associated with the MAPK signaling pathway and the citrullination of histone arginine residues. MSU and PMA could enhance the expression of MAPK signaling pathway-related proteins and induce citrullination of histones (Figure 3F–K). Therefore, we used MSU and PMA as inducers to induce NET formation in neutrophils, establishing a model for subsequent studies.

### 2.7. The Formation of NETs Can Be Prevented by Inhibitors of the MAPK Signaling Pathway

**Figure 4:**
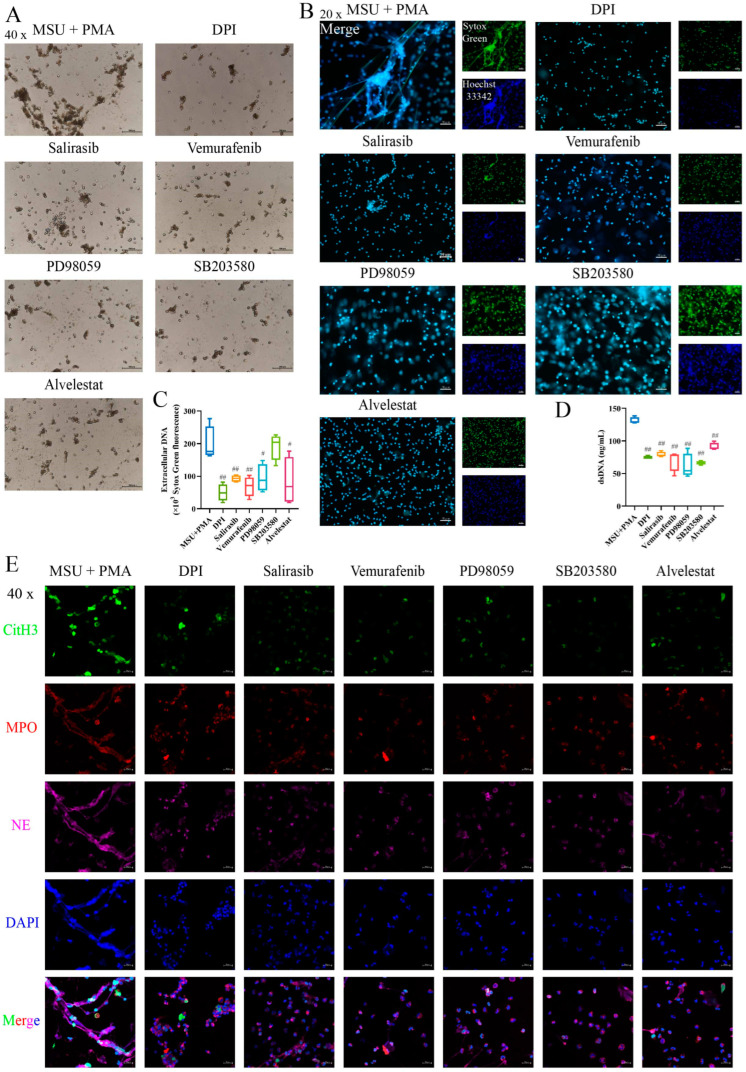
The formation of NETs can be prevented by inhibitors of the MAPK signaling pathway: (**A**) The morphological changes of cells treated with different selective inhibitors were observed under a light microscope. Scale bars, 100 μm. (**B**) Sytox Green dead cell nucleic acid dye (green) and Hoechst 33342 dye (blue) were used to observe the intervention of different inhibitors on NET formation. Scale bars, 50 μm. (**C**) The generation of NETs was quantified by Sytox Green dye at Ex/Em: 502/525 nm using a fluorescence microplate reader. (**D**) The cell supernatant dsDNA was measured with PicoGreen staining followed by a fluorescent microplate reader, *n* = 4. (**E**) Observation of NET release by laser confocal microscopy. Scale bars, 20 μm. DPI is a NOX inhibitor, Salirasib is a Ras inhibitor, Vemurafenib is a Raf inhibitor, PD98059 is an ERK inhibitor, SB203580 is a p38 MAPK inhibitor, Alvelestat is a NE inhibitor. (The same below.) The data were presented as mean ± SEM with ^#^ *p* < 0.05 or ^##^ *p* < 0.01 vs. MSU + PMA group. DPI, diphenyleneiodonium chloride; NOX, NADPH oxidase.

**Figure 5:**
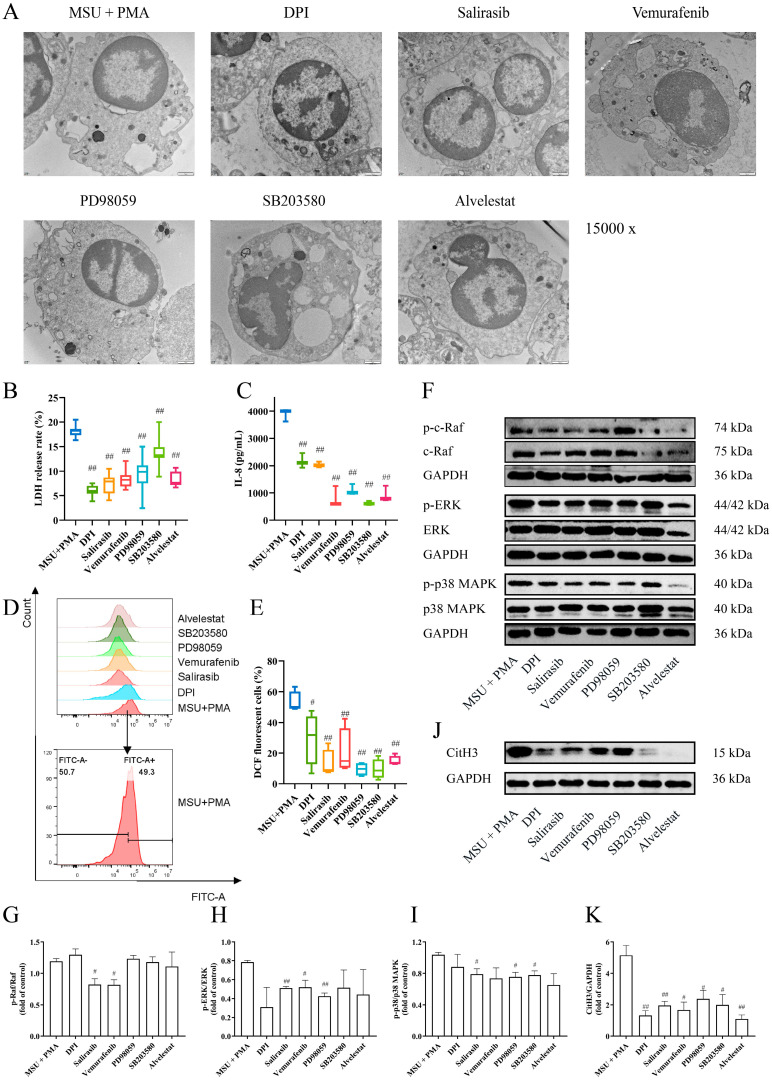
The NETs produced by MSU and PMA are related to ROS and MAPK signaling pathways: (**A**) TEM was used to observe the internal structure of NETs. Scale bars, 1 μm. (**B**) The LDH release was detected at dual wavelengths (490/600 nm), *n* = 8. (**C**) The cell supernatants were collected to determinate the IL-8 content, *n* = 3. (**D**) Flow cytometry was used to detect intracellular ROS. The black arrows represent the detailed presentation of the MSU + PMA group. (**E**) The three results of (**D**) were counted. (**F**–**K**) The key proteins of the MAPK signaling pathway and CitH3 in human neutrophils were detected by Western blotting. The data were presented as mean ± SEM with ^#^ *p* < 0.05 or ^##^ *p* < 0.01 vs. MSU + PMA group.

To further investigate the mechanism of MSU + PMA-induced NET formation, pathway inhibitors were employed. The light microscope observation revealed that cell morphology exhibited varying degrees of rollback following the addition of different inhibitors (Figure 4A). The results indicated that the formation of NETs was reduced to varying degrees, except for the p38 MAPK inhibitor (Figure 4B,C). The dsDNA levels in the cell supernatant showed similar trends (Figure 4D). Immunofluorescence results demonstrated various inhibitors were capable of suppressing the release of NET components, thereby inhibiting NET formation (Figure 4E). After treatment with different inhibitors, cell membranes were partially or fully preserved, some nuclei remained intact, and cells exhibited multiple vacuoles along with morphological changes in intracellular organelles (Figure 5A). Additionally, the inhibitors reduced LDH release (Figure 5B), IL-8 production (Figure 5C), and ROS production (Figure 5D,E). Notably, the p38 MAPK inhibitor SB203580 also showed a good inhibitory effect. To ascertain the involvement of the MAPK signaling pathway in NET formation, neutrophils treated with different inhibitors were analyzed for MAPK-related proteins via Western blotting. The results revealed a significant increase in CitH3 protein levels in the MSU + PMA group. Different inhibitors were able to reverse CitH3 expression to varying extents, indicating their role in inhibiting histone citrullination. Furthermore, the expression of proteins in the Raf-ERK-p38 MAPK signaling pathway was down-regulated in response to the inhibitors (Figure 5F–K).

## 3. Discussion

In recent years, the formation process of NETs has received extensive attention and research in metabolic diseases, tumors, and other diseases [43,44]. GA is a crystal-induced disease resulting from disturbances in purine metabolism, leading to the accumulation of uric acid and the deposition of MSU crystals in joint tissues [25]. NETs are considered one of the mechanisms underlying the pathogenesis of GA, and significant research efforts have been directed toward this area, but even so, there remain significant gaps. In this study, we have established a stable in vitro NET model for investigating crystalline diseases, especially GA, and to explore the underlying mechanisms involved.

It is widely recognized that NETs are reticular formations primarily composed of double-stranded DNA as their structural backbone, incorporating histones, MPO, NE, and antimicrobial peptides. Extracellular DNA is a major component of NETs, which can induce a pro-inflammatory cascade [45]. IL-8, a member of the CXC chemokine family, is a pro-inflammatory cytokine produced by immune cells or tissue cells, like macrophages and neutrophils. It contributes to the pathogenesis of diseases such as rheumatoid arthritis, GA, cancer, etc. [46,47]. In our study, 500 μM MSU alone did not appear to induce NET formation, but it significantly increased the levels of dsDNA, LDH, IL-8, and ROS. When combined with PMA, not only were these levels further elevated, but NET formation was also significantly enhanced (Figure 2 and Figure 3). This suggests that PMA amplifies the signaling response induced by MSU. Although multiple studies [18,38,40] have shown that different concentrations of MSU can induce NET formation, with most using concentrations between 200 μg/mL and 1 mg/mL and a few using 100 μg/mL [48,49] and 3 mg/mL [50], we chose a 500 μM (approximately 95 μg/mL) MSU concentration in this study. This choice aimed to better simulate the early pathological state of the disease and maintain consistency with the doses used in our previous studies [41,42]. Garcia’s study demonstrated that NET release in crystal-induced arthritis is dependent on the amount of crystals present (i.e., concentration-dependent) rather than the number of leukocytes or the type of crystals [31]. Therefore, the NET model induced by the combination of MSU and PMA is suitable for studying the pathological mechanisms of crystal-related diseases.

NE and MPO are typically found within the cytoplasm of neutrophils. However, during NETosis, NE translocases to the nuclear membrane, a necessary condition for chromatin condensation, while MPO binds to chromatin, further enhancing chromatin condensation [51,52,53,54]. Our study results indicated that Alvelestat (NE inhibitor) could effectively inhibit NET formation (Figure 3). This finding aligns with the work of Li et al. [55], who demonstrated that Alvelestat suppresses NETs and protects tissues from damage and inflammatory episodes. Additionally, the addition of MAPK signaling pathway inhibitors variably hindered the release of intracellular components, thereby suppressing NET formation. Chen et al. [56] have reported that PD98059, an ERK inhibitor, inhibits the formation of PMA-induced NETs, which is consistent with our findings (Figure 3). This suggests that MSU combined with PMA triggers NET formation through the Ras-Raf-ERK signaling pathway. We also observed marked vacuolization and chromatin decondensation in neutrophils, characteristic changes associated with NET release. Interestingly, the cell morphology showed varying degrees of reversion upon inhibitor intervention (Figure 3). The LDH assay results, which indicate membrane integrity, supported this observation. These findings further underscore that the combination of MSU and PMA triggers NET formation through the Ras-Raf-ERK pathway.

**Figure 6:**
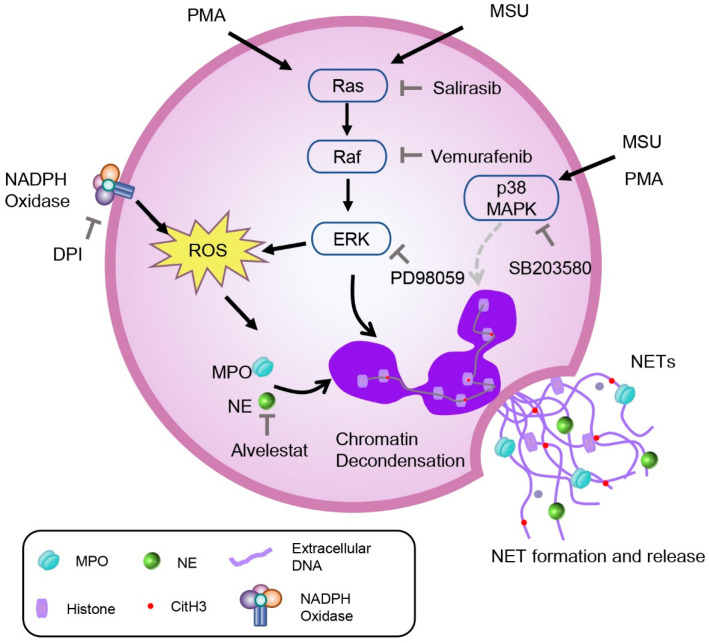
Schematic diagram of the mechanism of action of MSU and PMA to stimulate neutrophils to form NETs. Stimulation of neutrophils with MSU and PMA resulted in the activation of the Ras-Raf-ERK signaling pathway and generated ROS. MPO and NE released from cytoplasmic granules are transported into the nucleus, contributing to the unfolding of chromatin and citrullination of histones. Subsequently, the nuclear envelope ruptures, releasing chromatin and forming DNA-backbone networks (called NETs) outside the cell. After the use of pathway inhibitors, NET formation could be inhibited to a certain extent.

It is well known that ROS plays a crucial role in NETosis. Previous studies [20,57] have shown that the sources of these intracellular ROS are closely related to the activation of NOX. DPI, a non-specific inhibitor of flavin-dependent enzymes and a NOX inhibitor, significantly inhibits ROS produced upon PMA + MSU stimulation (*p* < 0.05). However, the role of ROS in MSU-induced NET formation is controversial. Davidsson et al. [40] argue that MSU-induced NET formation is independent of ROS production, proposing that ROS generation in neutrophils by MSU crystals occurs strictly intracellularly, not extracellularly. Some scholars believed that neutrophils could generate intracellular ROS in phagosomes or granules independently of phagocytosis [58]. This conclusion is based on the observation that colchicine markedly reduced MSU-triggered ROS generation without affecting ROS induced by the phagocytosis of *Staphyloccocus aureus*. However, other studies by Schorn et al. [18] and others [28,38] have shown that MSU crystals can induce NETosis in vitro through an ROS-dependent way. NETs also form in vivo, particularly during acute gouty flares and/or granuloma formation. Davidsson et al. [40] emphasized that MSU crystals stimulate NET formation in vivo within transmigrated neutrophils (from synovial fluids as well as skin chambers) without requiring ROS production. In contrast, Schorn et al. [18] highlighted that MSU crystals stimulate NET formation in neutrophils from the peripheral blood of healthy individuals in vitro in an ROS-dependent manner. This discrepancy may account for the inconsistent results of their studies. Interestingly, our results align with Schorn’s findings [18]. Our research demonstrated that suppressing the MAPK signaling pathway significantly reduces ROS production, indicating that MSU and PMA-induced NET formation depends on the Ras-Raf-ERK signaling pathway and ROS activation. In the study of the MAPK signaling pathway, we also introduced a p38 MAPK inhibitor. The results showed that the p38 MAPK inhibitor failed to suppress NET production and improve cell morphology but effectively inhibited IL-8 release. Wang et al. [59] found that inhibitors DPI (NOX inhibitor), U0126 (ERK inhibitor), and SB202190 (p38 MAPK inhibitor) could inhibit NaF-induced NET formation. Keshari et al. [60] also reported that SB202190 or U0126 inhibited the release of PMA-induced NETs. These findings are inconsistent with our results, potentially due to the use of different inhibitors. The inhibitor we used, SB203580, not only acts as a p38 MAPK inhibitor but also as an autophagy agonist. Studies [61,62] have shown that autophagy is also involved in PMA-induced NET formation. Therefore, in this study, even with the intervention of SB203580, NET release could not be completely inhibited. Further research is needed to explore the involvement of p38 MAPK in this combined model. All in all, these data indicated that the activation of the Ras-Raf-ERK signaling pathway, dependent on ROS, is crucial for MSU and PMA-induced NET formation (Figure 6).

## 4. Materials and Methods

### 4.1. Materials

PolymorphPrep^TM^ (Lot: 00121) was purchased from Axis-Shield (Serumwerk Bernburg AG, Bernburg, Germany). Phorbol 12-myristate 13-acetate (PMA, Lot: SLBX8889), monosodium urate (MSU, Lot: BC8R7559), and 4′,6-dia-midino-2-phenylindole (DAPI) were all from Sigma-Aldrich (St. Louis, MO, USA). Sytox Green was from Thermo Fisher Scientific (Waltham, MA, USA). Hoechst 33342, Triton X-100, ROS assay kit, and Lactate dehydrogenase (LDH) assay kit were all from Beyotime Institute of Biotechnology (Shanghai, China). Human IL-8 ELISA Kit was from Boster Biological Technology Co., Ltd. (Wuhan, China). Quant-iT™ PicoGreen^®^ dsDNA Reagent and Kits were from Thermo Fisher Scientific (Waltham, MA, USA). Diphenyleneiodonium chloride (DPI), Salirasib, Alvelestat, Vemurafenib, PD98059, and SB203580 were all from MedChemExpress (Monmouth Junction, NJ, USA).

PE anti-human CD16 antibody was from BD Bioscience (San Jose, CA, USA). FITC anti-human CD66b antibody was from Biolegend (San Diego, CA, USA). Anti-Histone H3 (citrulline R2 + R8 + R17) (CitH3) antibody, donkey polyclonal secondary antibody to rabbit IgG H&L (Alexa Fluor^®^ 488), donkey polyclonal secondary antibody to goat IgG H&L (Alexa Fluor^®^ 555), donkey polyclonal secondary antibody to mouse IgG H&L (Alexa Fluor^®^ 647) pre-adsorbed were all from Abcam (Cambridge, UK). MPO antibody was from R&D Systems (Minneapolis, MN, USA). NE antibody was from Santa Cruz Biotechnology Inc. (Santa Cruz, CA, USA). Anti-Raf, anti-p-Raf, anti-ERK1/2, anti-p-ERK1/2, anti-p38 MAPK and anti-p-p38 MAPK were from CST (Danvers, MA, USA). Anti-GAPDH was from Proteintech (Rosemount, IL, USA).

### 4.2. Experimental Design

The purified neutrophils were divided into 4 groups: A. Control group, B. MSU group, C. PMA group, and D. MSU + PMA group. Group A–D were stimulated with PBS, 500 μM MSU, 50 nM PMA, or 500 μM MSU + 50 nM PMA for 4 h, respectively.

Similarly, the purified neutrophils were divided into 7 groups: D. MSU + PMA group, E. MSU + PMA + DPI group, F. MSU + PMA + Salirasib group, G. MSU + PMA + Vemurafenib group, H. MSU + PMA + PD98059 group, I. MSU + PMA + SB203580 group, and J. MSU + PMA + Alvelestat group. Group D with 50 nM PMA + 500 μM MSU for 4 h. The E–J groups were pretreated with different inhibitors (DPI 10 μM, Salirasib 5 μM, Vemurafenib 5 μM, PD98059 10 μM, SB203580 10 μM, Alvelestat 10 μM) for 1 h before stimulation with MSU + PMA.

### 4.3. Extraction and Isolation of Neutrophils

Neutrophils were extracted from the peripheral blood of healthy volunteers using the PolymorphPrep^TM^ protocol. The brief steps were as follows: PolymorphPrep and an equal volume of fresh human peripheral blood containing EDTA were successively added into the centrifuge tube to maintain a clear interface. After centrifugation, the neutrophil layers were carefully extracted. Following red blood cell lysis, an appropriate volume of RPMI-1640 solution (containing 1% FBS, 1% bi-antibody, and 10 mmol/L HEPES) was added to ensure thorough mixing of the cells, which were then used for subsequent experiments.

### 4.4. Detection of Neutrophils Purity

Neutrophil purity was assessed using CD16 and CD66b markers [39,63]. Briefly, 1 × 10^6^ cells/mL cell suspension was stained with PE anti-human CD16 and/or FITC anti-human CD66b antibodies, with a blank control group included for comparison. The CD16+ and CD66b+ cell populations were analyzed using a Beckman flow cytometry (Beckman Coulter, Brea, CA, USA, CytoFlex). Neutrophils with a purity greater than 93% were utilized for subsequent experiments.

### 4.5. Observation of NETs Generation

The purified neutrophils were seeded onto L-polylysine-coated slides in 12-well plates containing and incubated at 37 °C under 5% CO_2_ for 0.5 h to allow adherence. After different treatments, the culture medium was discarded and pre-cooled 4% paraformaldehyde was added to each well for fixation for 20 min. Following two washes with PBS, Sytox Green and Hoechst 33342 were added sequentially to stain the cells for 10 min in the dark. Finally, a drop of antifade mounting medium was added to seal the slides after washing in PBS. The fibrous structure of extracellular DNA was observed under a fluorescence microscope (Carl Zeiss Microscopy, Gottingen, Germany, AXIO SCOPE.A1).

### 4.6. Quantification of NETs

NETs were quantified using Sytox Green [40,64,65]. Neutrophils (5 × 10^4^ cells/well) were seeded in 96-well transparent black plates (Corning, NY, USA). After completing the specified treatments, Sytox Green (final concentration 1 μM) was added to each well and incubated at 37 °C, 5% CO_2_ for 10 min. Meanwhile, control groups without Sytox Green (containing only cells) were set up for each group. The fluorescence intensity was measured at Ex/Em: 502/525 nm using a fluorescence microplate reader (BioTek, Winoski, VT, USA, Synergy H1), employing suspension measurement rather than bottom reading.

### 4.7. Detection of LDH Release

Neutrophils (5 × 10^4^ cells/well) were inoculated into 96-well plates. After the specified time, the samples were centrifuged using a high-speed centrifuge (Heal Force, Shanghai, China, Neofuge 15) at 400× *g* for 5 min. A total of 120 μL supernatant was collected, and 60 μL LDH working solution was added. The mixture was then incubated at 25 °C for 30 min under dark condition. LDH release rates (cell death rates) were measured using dual wavelengths (490/600 nm).

### 4.8. Determination of IL-8 and dsDNA

Neutrophils (1 × 10^6^ cells/mL) were inoculated into 6-well plates. After various treatments, the cell supernatant was collected and was centrifuged at 4 °C, 3000 rpm for 10 min. The level of IL-8 in the supernatant was detected according to the Human IL-8 ELISA Kit instruction. The dsDNA quantification is performed using the Quant-iT™ PicoGreen^®^ dsDNA Reagent and Kits. The supernatant from centrifuged samples is incubated with the PicoGreen at room temperature and protected from light for 5 min. The fluorescence intensity was measured at Ex/Em: 480/520 nm using a fluorescence microplate reader (BioTek, USA, Synergy H1).

### 4.9. Detection of ROS Generation

All cells were treated with DCFH-DA (1000:1) prepared in serum-free medium and incubated at 37 °C under 5% CO_2_ for 20 min, during which the cells were inverted and mixed every 3–5 min. Then, after different treatments of neutrophils, the cells were collected by centrifugation and washed with PBS. Beckman flow cytometry was used to detect intracellular ROS fluorescence intensity.

### 4.10. Observation of NETs Construction of by Laser Confocal Microscopy

We note that 2 × 10^5^ cells/well neutrophils were seeded in 12-well plates containing L-polylysine slides. After different treatments, the neutrophils were fixed with pre-cooled 4% paraformaldehyde for 20 min and permeabilized with 0.3% Triton X-100 for 10 min, followed by blocking with 5% BSA for 0.5 h. The samples were then stained with the following primary antibodies: rabbit Histone H3 antibody (1:400), goat MPO antibody (1:200), mouse NE antibody (1:200), and incubated in a wet box at 4 °C overnight. After washing with PBS, the samples were incubated for 1 h at room temperature with donkey polyclonal secondary antibody at a dilution of 1:1000. The samples were then incubated for 10 min with DAPI at a dilution of 1:10,000 in PBS, and then washed with PBS. Coverslips were mounted on glass microscope slides using an antifade mounting medium. Imaging of predetermined fields on the coverslip was performed using a laser scanning confocal microscope (SZX, Germany, LSM880). We observed the fluorescence intensity of CitH3, NE, MPO, and DAPI to evaluate the distribution of NET components.

### 4.11. Observation of NETs Microstructure by Transmission Electron Microscopy (TEM)

Differently treated neutrophils were fixed with 2.5% glutaraldehyde for 2 h at room temperature. After washing the samples twice with 0.1 M phosphate buffer and double-distilled water, they were fixed with a mixture of 1% osmic acid and 1.5% K_3_[Fe(CN)_6_] at 4 °C for 1 h. The samples were stained with 2% uranyl acetate solution overnight at 4 °C. After dehydrating with ethanol gradient, the samples were infiltrated with acetone, embedded in an embedding medium, and polymerized at 60 °C. They were then sectioned using an ultramicrotome (Leica, Wetzlar, Germany, UC7), attached to a copper grid, and observed for the microstructure of NETs using TEM (Hitachi Limited, Chiyoda, Japan, Hitachi H-7650).

### 4.12. Western Blotting

Neutrophils (2 × 10^6^ cells/well) were inoculated into 6-well plates. After different treatments, the cells were collected and lysed in 80 μL RIPA buffer containing 1% phosphatase inhibitor and protease inhibitor by ultrasonication at 4 °C for 15 min, and then the supernatant was collected by centrifugation at 12,000 rpm and 4 °C for 10 min. After denaturation, the samples were electrophoresed and separated in 10% polyacrylamide gel prior to being transferred onto PVDF membranes. Subsequently, the cropped PVDF membrane containing the target protein was blocked with 5% (*w*/*v*) non-fat milk in TBST buffer for 1 h at room temperature and incubated with corresponding primary antibodies at 4 °C for overnight. Raf, p-Raf, ERK1/2, p-ERK1/2, p38 MAPK and p-p38 MAPK were diluted to 1:1000 with TBST and GAPDH was 1:20,000. Then incubated with the anti-rabbit IgG or anti-mouse IgG (1:5000 dilution) for 1 h at room temperature. Some Western blotting experiments were replicated for probing the non-phosphorylated protein or the proximal band on the same PVDF membrane. The membranes were stripped with stripping buffer for 10 min and washed with TBST three times for 5 min before reprobing with another antibody. These target proteins were visualized by enhanced chemiluminescence system (Guangzhou Boluteng, Guangzhou, China). The optical density analysis of the protein bands was performed with the ImageJ 1.52a analysis program.

### 4.13. Statistical Analysis

The results were analyzed by two-tailed unpaired Student’s *t*-test using GraphPad Prism 8.0. The data were shown as mean ± SEM of at least three independent experiments. *p* < 0.05 was considered statistically significant.

## 5. Conclusions

Overall, this research established a model of NET formation induced by MSU and PMA. The changes in NETs were analyzed using various techniques, including fluorescence microscopy, laser confocal microscopy, flow cytometry, TEM, and Western blotting. Additionally, the selective inhibitors of Ras, Raf, ERK, NOX, and NE can prevent NET formation and inhibit the production of ROS, release of LDH, dsDNA, and IL-8 induced by MSU + PMA. These data indicated that the activation of a signaling pathway involving Ras-Raf-ERK, which was dependent on ROS, is crucial for the induction of NET formation by MSU and PMA. Given the involvement of NETs in multiple pathologies, our findings could potentially serve as molecular targets for the intervention and treatment of crystal-related diseases, especially for GA.

For future research directions, we will focus on knocking out key genes in the Ras-Raf-ERK pathway, including the use of knockout mice or cell transfection experiments, to better understand the specific roles of each component in the Ras-Raf-ERK pathway during NET formation. Simultaneously, in vivo experiments will be conducted to validate the results of in vitro studies and to further explore the systemic effects of NETs in crystal-related diseases. Further research can concentrate on developing small molecules or biologics targeting key components of the Ras-Raf-ERK pathway in order to reduce NET formation in patients with crystal-related diseases.

## Data Availability

Data will be made available on request.

## Abbreviations

CitH3, citrulline Histone H3; DPI, diphenyleneiodonium chloride; GA, gouty arthritis; LDH, lactate dehydrogenase; MPO, myeloperoxidase; MSU, monosodium urate; NE, neutrophil elastase; NETs, neutrophil extracellular traps; NOX, NADPH oxidase; PAD4, peptidyl arginine deiminase 4; PMA, phorbol 12-myristate 13-acetate; ROS, reactive oxide species; TEM, transmission electron microscopy.
